# Primary Adrenal Insufficiency During Lenvatinib or Vandetanib and Improvement of Fatigue After Cortisone Acetate Therapy

**DOI:** 10.1210/jc.2018-01836

**Published:** 2018-10-31

**Authors:** Carla Colombo, Simone De Leo, Marta Di Stefano, Guia Vannucchi, Luca Persani, Laura Fugazzola

**Affiliations:** 1 Division of Endocrine and Metabolic Diseases, IRCCS Istituto Auxologico Italiano, Milan, Italy; 2 Department of Pathophysiology and Transplantation, Università degli Studi di Milano, Milan, Italy; 3 Department of Clinical Sciences and Community Health, Università degli Studi di Milano, Milan, Italy

## Abstract

**Context:**

Two tyrosine kinase inhibitors (TKIs), lenvatinib and vandetanib, are often used to treat advanced radioiodine-refractory differentiated thyroid cancer (RAI-R DTC) and medullary thyroid cancer (MTC), respectively. Fatigue is a common adverse event during treatment with these and other TKIs and a common cause of drug discontinuation or dosage reduction.

**Cases Description:**

We evaluated the basal and stimulated adrenal function in 12 patients with advanced RAI-R DTC and MTC treated with lenvatinib or vandetanib, respectively. Ten patients complaining of fatigue showed a progressive ACTH increase with normal cortisol levels. Moreover, six of 10 patients had a blunted cortisol response after ACTH stimulation, thus confirming the diagnosis of primary adrenal insufficiency (PAI). The causal relationship between TKIs and PAI onset was also demonstrated by the repeated testing of adrenal function before and during treatment. Patients with PAI received cortisone acetate replacement therapy, with a substantial and prompt improvement in the degree of fatigue, as assessed by the Common Terminology Criteria for Adverse Events version 4.03, thus supporting the major impact of impaired adrenal function in the genesis of this adverse event.

**Conclusions:**

We show that the occurrence of PAI may be a common cause of fatigue during lenvatinib and vandetanib treatment, and we therefore recommend testing adrenal function for a prompt start of replacement therapy to avoid treatment discontinuation, dosage reduction, and potentially severe PAI complications.

## Teaser

Primitive adrenal insufficiency was diagnosed in patients on lenvatinib or vandetanib and prompt improvement in the degree of fatigue was documented upon cortisone acetate replacement treatment.

Two tyrosine kinase inhibitors (TKIs), lenvatinib and vandetanib, are often used to treat advanced radioiodine-refractory differentiated thyroid cancer (RAI-R DTC) and medullary thyroid cancer (MTC), respectively. Lenvatinib inhibits both angiogenesis, acting on VEGFR 1-3, FGFR 1-4, and PDGFR *α*, and carcinogenesis, acting on RET and c-KIT. Vandetanib works on VEGFR 1-3 and EGF-R and on RET, which is the oncogene responsible for MTC. Several adverse events (AEs) have been reported in almost all treated patients, largely overlapping between the two compounds and including hypertension, fatigue, diarrhea, and weight loss. They led to treatment discontinuation in 14.2% of patients on lenvatinib (SELECT study) and 12% of patients on vandetanib (ZETA study). Unexplained fatigue or asthenia is present in 59% and 24% of patients treated with lenvatinib and vandetanib, respectively, and it is one of the most common causes of treatment discontinuation (1, 2). Despite recommendations to have patients to take TKIs in the evening and do physical exercise, to monitor electrolytes and TSH levels, and to maintain hydration and adequate food intake (3), in most cases the symptom persists and dosage reductions are needed.

Information on adrenal function during TKI treatment is scanty. An increased prevalence of subclinical hypocortisolism in patients on imatinib was reported (4), whereas an elevation of cortisol was found in 14 patients treated with vandetanib, although the possible diagnosis of hypercortisolism was ruled out by the free urinary cortisol in the lower part of normal range (5).

## Methods

After obtaining written informed consent, we evaluated 12 consecutive patients with advanced RAI-R DTC and MTC during treatment with lenvatinib (n = 7) and vandetanib (n = 5) and followed up at a single institution. The clinical features of the patients are reported in Table 1. Fatigue was assessed by the Common Terminology Criteria for Adverse Events (CTCAE) version 4.03. None of the patients complained of fatigue before the start of lenvatinib treatment (grade 0). We performed in all patients, at different stages of treatment, a basal evaluation of adrenal function during the morning (8:00 to 9:00 am) and in a fasting state. Patients on cortisone acetate (CA) always received the treatment after blood sampling. Serum and urinary free cortisol and ACTH were assayed with the Elecsys^®^ cortisol and ACTH immunoassays (Roche Diagnostics, Mannheim, Germany). All patients also underwent a 250-µg ACTH stimulation test, which is considered the gold standard to establish the diagnosis of primary adrenal insufficiency (PAI) (6). The morning of the ACTH test, we assessed at baseline salivary cortisol, 24-hour urinary free cortisol, and adrenal autoantibodies. Salivary cortisol was assayed with liquid chromatography–tandem mass spectrometry with the Waters Xevo TQ MS system (Waters, Milford, MA; normal values >0.265 μg/dL), and adrenal autoantibodies were determined by an indirect immunofluorescence assay with precoated primate adrenal slides (Astra Formedic, Lessolo, Italy). Only in patient 7 was adrenal function evaluated before the start of TKI treatment.

**Table 1.** Clinical Features of the Patients Treated With Lenvatinib and Vandetanib

Patient/Sex	Age at Diagnosis/Age at TKI Start (y)	Tumor Histotype	TNM/AJCC Stage	ECOG Status	Treatment (Dose, mo)	Adverse Events (Grade)^*a*^	Disease Status
1/F	63/74	PDTC	pT3NXM0/II	1	Lenvatinib (20 → 10 mg, 21)	Hypertension (3), fatigue (2), diarrhea (1), weight loss (1), anorexia (1), proteinuria (2), reversible posterior leukoencephalopathy (3)	PR
2/F	42/58	FTC	pT2NXM0/I	0	Lenvatinib (20 mg, 21)	Hypertension (3), fatigue (2), diarrhea (2), anorexia (1), palmar-plantar erythrodysesthesia syndrome (1), stomatitis (1), hemorrhoids (3)	SD
3/M	51/58	PTC Tall cell	pT4aN1aM0/ I	0	Lenvatinib (24 mg, 13)	Fatigue (2), diarrhea (1), weight loss (1), anorexia (1), proteinuria (1), palmar-plantar erythrodysesthesia syndrome (1)	PR
4/F	33/75	CPTC	N/A	1	Lenvatinib (10 mg, 13)	Hypertension (3), fatigue (1), diarrhea (2), stomatitis (3), nausea (1), hemorrhoids (2)	PR
5/F	71/72	CPTC	pT3NXM0/II	1	Lenvatinib (10 mg, 15)	Hypertension (2), fatigue (2), diarrhea (2), anorexia (1), skin ulceration (2), arthralgia (1), hoarseness (1), dysgeusia (1)	PR
6/M	67/68	FTC	pT4bNXM1/IVb	0	Lenvatinib (20 mg, 9)	Hypertension (3), stomatitis (2), dysgeusia (1)	SD
7/M	54/66	CPTC	pT4aN1MX/I	0	Lenvatinib (20 mg, 5)	Fatigue (2), weight loss (1), hypertension (3), anorexia (1)	N/A
8/M	46/63	sMTC	pT4N1bM1/II	1	Vandetanib (100 mg, 44)	Fatigue (2), diarrhea (3), anorexia (1)	SD
9/F	8/9	MEN2B	pT4aN1b/II	0	Vandetanib (200 mg, 118)	Fatigue (2), weight loss (1)	SD
10/M	9/20	MEN2B	N/A	0	Vandetanib (300 → 150 → 100 mg, 43)	Fatigue (1), diarrhea (1), QTc prolongation (2)	SD
11/F	21/28	sMTC	pT3N1bM1/II	0	Vandetanib (300 mg, 10)	Fatigue (2), diarrhea (1), maculopapular rash (1)	PR
12/F	72/76	sMTC	pT2N1b/II	1	Vandetanib (300 → 150 → 100 mg, 36)	Anorexia (1), QTc prolongation (2)	PR

Required dosage reductions are reported.

Abbreviations: AJCC, American Joint Committee on Cancer; CPTC, papillary thyroid cancer classic variant; F, female; FTC, follicular thyroid cancer; M, male; MEN2B, multiple endocrine neoplasia 2B; N/A, not available; PDTC, poorly differentiated thyroid cancer; PR, partial response; PTC, papillary thyroid cancer; SD, stable disease; sMTC, sporadic MTC; TKI, tyrosine kinase inhibitors; TNM, tumor size, lymph nodes affected, metastases.

^a^ Severity grade was measured according to the CTCAE, version 4.03; the degree of fatigue reported is intended before the start of CA treatment.

## Results

**Figure 1.**
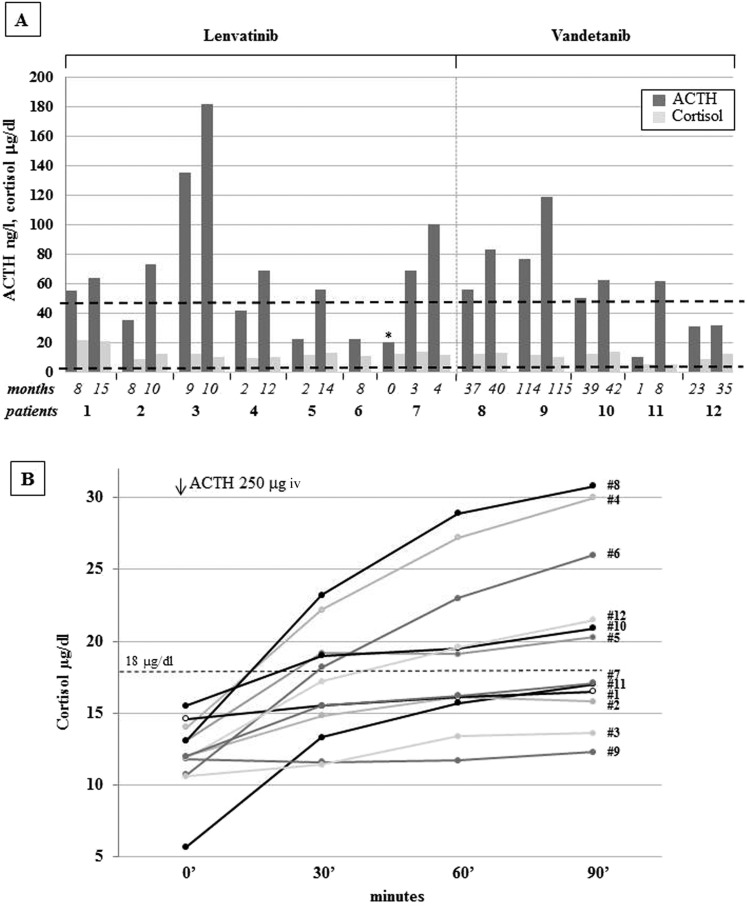
(A) Cortisol (μg/dL) and ACTH (ng/L) levels in the patients treated with lenvatinib (n = 7) and vandetanib (n = 5) according to treatment duration. In particular, the first adrenal function evaluation and the last assessment before the start of cortisone acetate replacement are reported. Interestingly, for patient 7, the evaluation of adrenal function was available before the start of the lenvatinib treatment (asterisk), and the two following evaluations without cortisone acetate therapy are also reported. The lowest normal cortisol level (5 μg/dL) and the highest normal ACTH level (50 ng/mL) are reported in the figure. (B) Cortisol levels at baseline and at 30, 60, and 90 minutes after infusion of 250 mμg ACTH in the 12 patients. CA replacement treatment was started in patients 1, 2, 3, 7, 9, and 11. Peak cortisol levels <500 nmol/L (18 μg/dL, dotted line) at 30 or 60 minutes indicate adrenal insufficiency.

**Figure 2.**
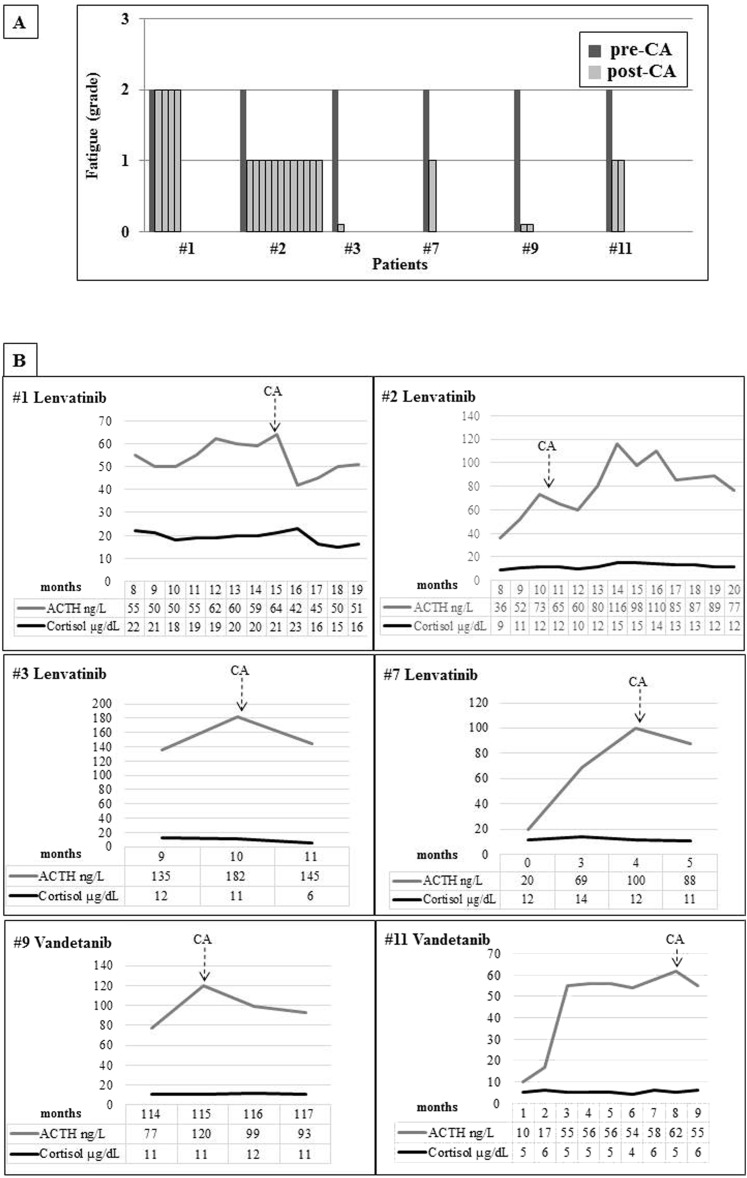
(A) Degree of fatigue according to the CTCAE (version 4.03) in patients before treatment with CA and at each control after the start of the replacement therapy. (B) ACTH and cortisol levels during TKI treatment in patients started on CA therapy. The basal evaluation of the adrenal function was always assessed during the morning (8:00 to 9:00 am) and in a fasting state.

Ten out of the 12 treated patients complained of fatigue (six on lenvatinib and four on vandetanib). Only these 10 patients showed a progressive increase in ACTH levels with normal cortisol concentrations, a combination of findings consistent with incipient PAI (Fig. 1A). In patient 7, ACTH levels rose from 20 ng/L at baseline to 100 ng/L during the first months of lenvatinib treatment. The 24-hour urinary free cortisol levels were in the lower part of the normal range in all patients (39.73 ± 6.0 μg, normal levels 17.0 to 136.0 μg); mean morning salivary cortisol levels were 0.31 ± 0.08 μg/dL, below the normal range in six of 10 patients, and adrenal autoantibodies were negative in all patients (Table 2). Moreover, six of the 10 patients complaining of fatigue showed an impaired response of cortisol upon ACTH infusion, thus confirming the PAI diagnosis (Fig. 1B) (6). Interestingly, patient 3 experienced an adrenal crisis immediately after the ACTH test, with prompt resolution upon hydrocortisone administration, further supporting the diagnosis of adrenal insufficiency. Whole body CT or whole body fluorine-18–2-fluoro-2-deoxy-d-glucose positron emission tomography and abdominal ultrasound ruled out adrenal metastases or adrenal gland alterations in all cases. Hepatic and renal functions, glycemia, and hemoglobin were in the normal range in all cases, and none of the patients was on treatment with drugs potentially leading to fatigue (*e.g.,* antipsychotics). Renin and aldosterone were not measured because all patients were on interfering antihypertensive treatment because of a known TKI side effect. Nevertheless, the finding of normal electrolyte levels in all patients is consistent with conserved mineralocorticoid function. In patients with RAI-R DTC, l-thyroxine dosage was titrated to maintain TSH levels below the normal limit (0.01 to 0.1 mU/L), either before or during lenvatinib treatment. In patients with advanced MTC, TSH levels were in the normal range (0.5 to 2 mU/L). No significant correlation between TKI dosage and the degree of fatigue was observed (data not shown). Patients with fatigue grade 2 and those with an established diagnosis of PAI (1, 2, 3, 7, 9, and 11) received CA replacement at the standard dosage (25 to 35 mg/day). All patients but 1 reported substantial improvements in fatigue immediately after the first days of treatment (to grade 1 or 0 in three or two cases, respectively). No symptom relief was reported by patient 1, who had cortisol levels stably in the high normal range (Fig. 2A). This patient developed minor depression during TKI treatment, and it is likely that the related manifestations could have masked the beneficial effects of CA replacement. As in typical patients with PAI, ACTH levels remained elevated during replacement treatment (Fig. 2B), and the CA dosage was adjusted based on clinical response (6).

**Table 2.** Urinary Free Cortisol, Morning Salivary Cortisol Levels, and Adrenal Autoantibodies in the Cohort

	Morning Salivary Cortisol (>0.265 μg/dL)	24-h UFC (17–136 μg/24 h)	Adrenal Autoantibodies	Response to ACTH Test (>18 μg/dL)	Fatigue (CTCAE 4.03)
**1**	**—**	**39.5**	**Negative**	**Impaired**	**Yes**
**2**	**0.218**	**30.9**	**Negative**	**Impaired**	**Yes**
**3**	**0.236**	**45.2**	**Negative**	**Impaired**	**Yes**
4	0.325	—	Negative	Normal	Yes
5	—	44.3	Negative	Normal	Yes
6	0.272	—	Negative	Normal	No
**7**	**0.246**	**—**	**Negative**	**Impaired**	**Yes**
8	0.473	47.1	Negative	Normal	Yes
**9**	**0.241**	**—**	**Negative**	**Impaired**	**Yes**
10	0.364	35.4	Negative	Normal	Yes
**11**	**0.192**	**35.7**	**Negative**	**Impaired**	**Yes**
12	0.261	—	Negative	Normal	No

Patients reported in bold have an impaired response to ACTH stimulation test and consistently low morning salivary cortisol levels.

Abbreviation: UFC, urinary free cortisol.

## Discussion

These data show the frequent occurrence of PAI after the first months of treatment with lenvatinib or vandetanib in patients with advanced RAI-R DTC or MTC. PAI is a likely cause of the chronic severe fatigue reported by several patients and may represent a life-threatening condition under stress. Importantly, replacement treatment with CA is associated with substantial relief of this major adverse event. Because assessment of the adequacy of replacement with glucocorticoids cannot rely on morning ACTH levels, fine-tuning of daily cortisone acetate dosage should be done only through a careful and complete clinical assessment, including evaluation of fatigue (7).

Adrenal toxicity, represented as cortical hemorrhage or necrosis, was seen in rats and monkeys treated with sunitinib (8), possibly because of the reduced density of capillary networks seen in some endocrine organs upon administration of anti-VEGF compounds (9, 10). Although anti-VEGF treatments often lead to the development of hypothyroidism in mice (10) and humans, scanty and controversial data are available on adrenal function (4, 5), probably because of the existence of compensatory mechanisms or misdiagnosis. Indeed, thyroid function is always evaluated during TKI administration, but adrenal function testing is not recommended. Our data strongly suggest that appropriate testing of adrenal function should be included in the follow-up of patients treated with lenvatinib or vandetanib to prevent the onset or worsening of fatigue and other severe consequences of PAI. The limitations of our study include the limited number of subjects, the lack of evaluation of adrenal function before TKI began in all patients, and the lack of a control group. Nevertheless, although for most patients adrenal function was tested at various times after the start of TKI treatment, a clear causal relationship between TKI and PAI onset was seen in patient 7, whose adrenal function was repeatedly tested before and during lenvatinib treatment. Moreover, the substantial improvements in fatigue observed in patients during CA treatment give strong support to our conclusions, which certainly need to be confirmed in a controlled trial. We therefore recommend that patients on lenvatinib or vandetanib who experience notable fatigue should undergo adrenal function testing and that replacement treatment should be started in those with a PAI diagnosis, to avoid unwanted TKI discontinuation or reduction.

## Abbreviations:

AE: adverse event
CA: cortisone acetate
CTCAE: Common Terminology Criteria for Adverse Events
MTC: medullary thyroid cancer
PAI: primary adrenal insufficiency
RAI-R DTC: radioiodine-refractory differentiated thyroid cancer
TKI: tyrosine kinase inhibitor
